# Seronegative *Myasthenia Gravis* with Concomitant SARS-CoV-2 Infection in a Dog

**DOI:** 10.3390/vetsci9070318

**Published:** 2022-06-24

**Authors:** Mihai Musteata, Denis-Gabriel Borcea, Andreea Despa, Raluca Ștefănescu, Larisa Ivănescu, Luminița Diana Hrițcu, Radu Andrei Baisan, Radu Lăcătuș, Gheorghe Solcan

**Affiliations:** 1 Neurology Service, Faculty of Veterinary Medicine, Ion Ionescu de la Brad Iași University of Life Sciences, 700489 Iași, Romania; borceadega@yahoo.com (D.-G.B.); andreeadespa@yahoo.com (A.D.); raluca.stef@yahoo.ro (R.Ș.); 2 Parasitology Service, Faculty of Veterinary Medicine, Ion Ionescu de la Brad Iași University of Life Sciences, 700489 Iași, Romania; larisssa81@yahoo.com; 3 Internal Medicine Clinic, Faculty of Veterinary Medicine, Ion Ionescu de la Brad Iași University of Life Sciences, 700489 Iași, Romania; lumidih@uaiasi.ro (L.D.H.); gsolcan@uaiasi.ro (G.S.); 4 Radiology Unit, Faculty of Veterinary Medicine, Ion Ionescu de la Brad Iași University of Life Sciences, 700489 Iași, Romania; baisan.andrei_mv@uaiasi.ro; 5 Faculty of Veterinary Medicine, University of Agricultural Sciences and Veterinary Medicine Cluj-Napoca, 400372 Cluj-Napoca, Romania; rlacatus2003@yahoo.com

**Keywords:** seronegative *myasthenia gravis*, dog, coronavirus

## Abstract

**Simple Summary:**

Myasthenia gravis is a disorder of neuromuscular transmission affecting neuromuscular junction. Both congenital and acquired forms are described in dogs. Usually, the acquired form might be induced by an autoimmune attack against specific acetylcholine receptors. Viral infection is incriminated to be a trigger for myasthenia gravis occurrence and, in a limited number of papers, infection with SARS-CoV-2 was found to be associated with myasthenia gravis expression in humans. In this case report, we describe a 2-year old crossbred, female dog case of seronegative generalized myasthenia gravis and concomitant SARS-CoV 2 infection due to close exposure to an infected owner. Despite the viral particles were not identified in our dog, the presence of anti SARS-CoV 2 antibodies and the epidemiological context were highly suggestive for a new recent infection with SARS-CoV 2. Our data complete the limited number of reports which document the SARS-CoV 2 passage from owners to pets and underline the role of serological screening for specific anti-SARS-CoV 2 antibodies as an indirect marker of infection especially when the viral detection fail to PCR test.

**Abstract:**

*Myasthenia gravis* (MG) is a disorder of neuromuscular transmission affecting the neuromuscular junction. The majority of cases involve an autoimmune attack against AChR, but a limited number of patients are seronegative for AChR antibodies. Viral infection is incriminated as a trigger for MG occurrence, and in a limited number of reports, infection with SARS-CoV-2 was found to be associated with MG expression in humans. In this report, we describe case of seronegative generalized MG in a 2-year-old crossbred female dog associated with SARS-CoV-2 infection due to close exposure to an infected owner.

## 1. Introduction

*Myasthenia gravis* (MG) is a disorder of neuromuscular transmission affecting the neuromuscular junction. MG is clinically expressed in three forms. In the focal one, only the cranial nerves are involved, and the clinical signs are limited to regurgitation (as a result of esophageal dilatation), dysphagia (as a result of pharyngeal dysfunction), voice change (as a result of laryngeal paralysis), or multiple cranial nerve abnormalities in the absence of generalized muscle weakness (dysphagia, megaesophagus). Animals with the generalized form elicit a generalized weakness associated or not with esophageal or pharyngeal dysfunction [1]. This clinical form evolves in an episodic frame and total recovery appears after rest. The fulminant form is characterized by a sudden onset of esophageal dilatation, rapid progression to quadriparesis, respiratory failure, and high mortality [2].

MG is caused by either a deficiency or functional disorder of the nicotinic acetylcholine receptor (AChR) in congenital MG or by an autoimmune attack against AChRs, resulting in the depletion of receptors in acquired MG [1,2], but autoantibodies targeting muscle-specific kinase (MUSK) also are reported. MG may be associated with other autoimmune [3], endocrine, and neoplastic disorders [4]. In humans, acquired MG immunological cross-reactivity between AChR peptides and viral/microbial proteins has been reported, and some viruses are known to be triggers for MG expression [5,6,7,8].

In this paper, we describe a case of the generalized form of seronegative MG in a dog associated with SARS-CoV-2 infection.

## 2. Case Description

**Figure 1:**
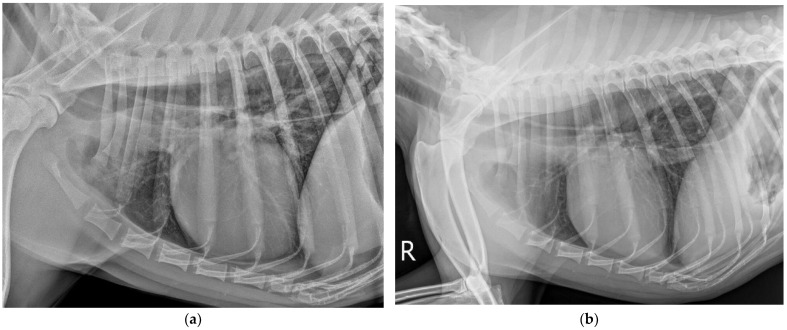
(**a**) Lateral thoracic X-ray at the first consultation. Note the diffuse interstitial pulmonary pattern visible in cranial and diaphragmatic lung lobes, along with a bronchial pattern in the diaphragmatic lobes and esophageal gas distension. (**b**) Lateral thoracic X-ray upon admission to our service (4 weeks later). The pulmonary pattern and esophageal gas distension are visible.

A 2-year-old crossbred, 22 kg, neutered, female dog was presented to the neurology service at the Veterinary Teaching Hospital Iasi with signs of progressive weakness. The clinical signs started 4 weeks prior to presentation, 11 days after the owner was diagnosed with SARS-CoV-2 virus and quarantined. The initial clinical signs consisted of mild dysphagia and a tendency to avoid physical activity. After two weeks of quarantine, once the dog’s regular physical activity was restored, the owners remarked that the dog was not able to walk properly for longer distances and reported severe dysphagia associated with regurgitation episodes. The dog was presented to a general practitioner vet and a complete blood test and thoracic X-rays were performed. On the thoracic X-ray, a diffuse interstitial pulmonary pattern was visible in cranial and diaphragmatic lung lobes, along with a bronchial pattern in the diaphragmatic lobes on the right lateral chest image. The cardiac silhouette appeared normal in shape and size without dorsal displacement of the trachea. A radiolucent tubular area was visible in the dorsal third of the thorax extending from the thoracic inlet towards the diaphragm, consistent with esophageal gas distention (Figure 1a). On the ventro-dorsal view, the diaphragmatic lung lobes had a diffuse interstitial peri-bronchial pattern. The cardiac silhouette appeared normal. The esophageal distension was not visible in this view. On cell blood count, no changes were observed for red cells and leukocytes, but trombocytemia (18 × 10^3^/µL, normal range 211–621 × 10^3^/µL) was noticed. Serum biochemistry was unremarkable, with the exception of alanineaminotransferase (ALT), which was increased (154 UI/L, normal range 10–50 UI/L). A blood smear was analyzed and *Babesia gibsoni* infestation was observed. Doxicilyne (7–10 mg/Kg, q 12 h, Doxiciclina, Antibiotice Iasi, Iasi, Romania) and atovaquone (13.3 mg/kg per-os, q 8 h) therapy was started for 2 weeks, after which the blood smear was negative for *Babesia*. However, the patient was not able to sustain a normal walk, even for short distances (especially on the posterior limbs). To exclude Lyme disease, a blood sample was submitted for *Borellia* antibodies measurement (ELISA), and the results were in normal limits (IgG 1.1 VE (negative < 8 VE) and IgM 4.8 VE (negative < 8 VE), see Supplementary Figure S1).

Because of the increased fatigability, the dog was referred to our service for a neurological examination.

At the presentation, an exercise-induced collapse (the dog was able to sustain a normal walk for less than 20 m) was observed. Both conscious proprioception and spinal reflexes were normal at the beginning of neuroexamination but diminished and became absent after multiple examinations. Cranial nerves were normal, with the exception of the blink reflex, which disappeared after few stimulations of both internal and external canthus. The consciousness level was normal. The perineal reflex, urinary and feces continence, voluntary movements of the tail, and nociception were preserved.

The rectal temperature was 38.8 °C, the heart rate (HR) was 165 beats/min, and the respiratory rate was 32 breaths/min.

The neuroanatomical localization was consistent with a generalized lower motor neuron condition. A mild to moderate score (4/5) according to the Muscle Weakness Scoring System [9] was calculated.

Blood work revealed a slight increase in hematocrit (57.82%, normal range 37–55%) and an increased number of red blood cells (8.55 × 10^6^ cells/uL, normal range 5.5–8.5 × 10^6^ cells/µL) and hemoglobin (19.2 g/dL, normal range 12–18 g/dL). The rest of the parameters for cell blood count were in the normal range. Multiple blood smears were submitted for cytological examination. The blood cytology was unremarkable and no hemoparasites were observed. A full biochemistry profile (Comprehensive Diagnostic Kit, ABAXIS, Griesheim, Germany) was performed. Increased ALT (475 UI/L, normal range 10–50 UI/L) and borderline albumin (4.4 g/dL, normal range 2.4–4.4 g/dL) associated with a slightly decreased level of globulin (2.0 g/dL, normal range 2.3–5.2 g/dL) were seen. Thyroid function was found to be normal. Canine TSH was normal (<0.25 ng/mL), T4 was normal (1.55 ug/dL, normal range 1–4 ug/dL), cholesterol was 228.6 mg/dL (normal range 135–278 mg/dL), and triglycerides were 36 mg/dL (normal range < 150 mg/dL). Canine C reactive protein (cCRP) was normal (19.1 mg/L, normal range < 20 mg/L), and no changes were observed when blood gases (I Stat profile PrimePlus) and urine were analyzed.

Due to the dog’s close contact with a COVID-19-infected owner, a blood immunofluorescence antigen rapid test (Standard F COVID-19 Ag FIA, SD Biosensor, Cheongju-si, Korea; F200 analyzer SD, Cheongju-si, Biosensor Korea) and a PCR test (nasal, oral, and rectal swabs were collected) were performed. The BioMagPure 12 plus Automated bench-top nucleic acid extraction system from Biosan—Medical-Biological Research and Technologies was used for RNA extraction. The BioMagPure Viral RNA extraction kit was used for extracting viral RNA from plasma or serum. The CFX96 Dx Real-Time PCR Detection System for In Vitro Diagnostics (IVD) was used for real-time PCR diagnosis. The kit STAT—NAT COVID-19 MULTI REF 1N036 was used for real-time PCR diagnosis. It is a lyophilized mix for the qualitative detection of novel coronavirus SARS-CoV-2 in real-time PCR. The STAT—NAT COVID-19 MULTI kit is an in vitro diagnostic medical device and it has been designed for professional use in specialized clinical and research laboratories. This kit is based on RT-PCR testing and Sentinel STAT-NAT (Stabilized Amplification Technology–Nucleic Acid Testing) technology. The kit consists of one optimized freeze-dried reaction mixture (96 reactions total) simultaneously targeting two regions of the specific SARS-CoV-2 RdRP Gene and Orf1b genes. All probes for the specific regions are labeled with FAM fluorophore, allowing for fast and simple evaluation of results. Primers and probes specific for a housekeeping gene labeled with HEX fluorophore are present in each reaction and they are used as the endogenous internal control (IC). Both tests (immunofluorescence and PCR) failed to identify the SARS-CoV-2 virus.

On radiography, in the right lateral thoracic image, a normal pulmonary pattern was visible. The cardiac silhouette appeared with a normal shape and size, with a VHS (vertebral heart score) of 9.9 v and normal aspect and position of the trachea. The vascular structures were normal. A radiolucent tubular area was visible in the upper third of the chest extending from the cranial thoracic inlet to the diaphragm, consistent with esophageal gas distension (Figure 1b).

No abnormalities were noticed on the abdominal ultrasonography (LogiqV5 ultrasound machine equipped with a 4–8-MHz phased-array probe, General Electric Medical Systems, Wuxi, China) and on 5 min six-leads ECG examination (ECG; PolySpectrum 8 V ECG machine, Neurosoft, Ivanovo, Russia). The blood pressure was assessed non-invasively in the coccygeal artery with the dog positioned in lateral recumbency, using an oscillometric veterinary device equipped with a size-suitable cuff (VET HDO, S  +  B medVet, D2 cuff, Babenhausen, Germany). The mean value of five artefact-free measurements evaluated through the dedicated arterial pulse graphic was used for analyses [10]. After taking the blood pressure, the patient was considered as normotensive, with a systolic blood pressure (mean (m) ± standard deviation (SD)) of 152  ±  9 mmHg and diastolic blood pressure (m  ±  SD) of 85  ±  2 mmHg.

The blood creatine kinase and cholinesterase levels were checked and found to be in normal limits (69.7 UI/L, normal range 10–200 UI/L [11] and 6168.5 U/L, normal range 3164–8164 U/L [12], respectively).

**Figure 2:**
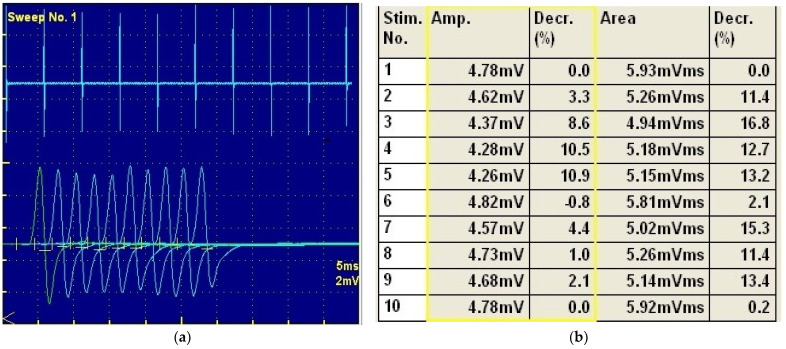
(**a**) Repetitive nerve stimulation test and the fatigability of the synaptic transmission. (**b**) There is an obvious 10% reduction in the amplitudes of the fourth compound muscle action potential compared to the first.

The electrophysiological tests were performed using the Neuropack S, MEB 9400 K electrodiagnostic System (Nihon Kohden, Tokyo, Japan) under general anesthesia (Propofol 2 mg/kg i.v. route, Propofol Fresenius, Fresenius Kabi, Germany; and isoflurane 0.5–2%, Anesteran, Rompharm, Romania). Electromyography tests were normal for both the pelvis and thoracic limb musculature and motor nerve conduction velocity measurements performed for the peroneal, tibial, and radial nerves based on recordings from the plantar, palmar interosseous, and extensor carpi ulnaris muscles, respectively. A decrement in the repetitive stimulation test was obtained for tibial and radial nerves (Figure 2a,b). The findings were suggestive of a neuromuscular junction disorder.

Based on clinical examination, X-ray findings, and electrodiagnostic tests, *myasthenia gravis* was presented as the main diagnostic hypothesis, and a serum sample was submitted for testing the anti-acetylcholine receptor antibody or AChR antibody. The result was negative for circulating AChR antibodies (cell line TE671: 50, normal < 400; cell homogenate: 50, normal < 300) (see Supplementary Materials).

A neostigmine challenge was performed the next day. Neostigmine methylsulfate (Miostin, Zentiva, Romania) 0.02 mg/kg s.c. route followed by Atropine (Atropinum sulphuricum, Romvac, Romania) (0.005 mg/kg s.c. route) were administered. The response to neostigmine was rapid and consistent (see Supplementary Video S4), and no serious side effects were noticed, with the exception of a transient epiphora at 10 min following the anti-cholinesterasic agent administration.

Starting that day, therapy with piridostigmine 1 mg/kg, per os, q 8 h (Mestinon, Labiana Pharmaceuticals, S.L, Barcelona, Spain); metoclopramide 0.5 mg/kg, per os, q 8 h (Metoclopramid, Terapia, Romania); and omeprazole 0.75 mg/kg, per os, q 24 h (Omeprazol, Terapia, Romania) was initiated.

Ten days after the patient’s presentation to our service, an increase in serum cCRP was observed (27.5 mg/dL at 7 days, 48.3 mg/dL at 9 days, 67.5 mg/dL increase 12 days), but normal CBC. Prednisolone therapy was instituted at an anti-inflammatory dosage (0.5 mg/kg/day, per os, Prednicortone, Le Vet Beheer B.V., Oudewater, Holland) for two weeks. After the initiation of prednisolone therapy, the CRP decreased below 10 mg/dL and the prednisolone was tapered to 0.5 mg/kg once every two days for another 2 weeks.

Eight weeks after the first presentation to our service (twelve weeks after the first clinical signs), a blood sample was submitted to an external lab for the specific SARS-CoV-2 antibody profile. Nucleocapsid protein, spike S1 subunit receptor binding domain (RBD), S2 unit of spike (Spike S2), envelope protein, angiotensin-converting enzyme 2, papain-like protease, and anti-SARS-CoV-2 IgG, IgA, and IgM antibodies were investigated and quantified by the microblot technique. The results show the presence of anti-SARS-CoV-2 IgG, IgA, and IgM antibodies (Table 1). The highest values were obtained for IgM, especially for Spike 2, envelope protein, angiotensin-converting enzyme 2, and papain-like protease.

Two months after the initiation of combined anti-cholinesterasic agent and glucocorticoid therapy, the dog condition was stable, the effort tolerance was normal, and no cranial nerves deficits were observed, with the exception of a decreased blink reflex when the external canthus was stimulated. The radiological aspect consistent with megaesophagus was still present.

## 3. Discussion

To the best of our knowledge, this is the first report of a dog with seronegative MG associated with SARS-CoV-2 infection due to close exposure to an infected owner. To date, there are limited reports describing dogs acquiring infection in households with SARS-CoV-2-infected humans [13,14] or in which the evidence of transmission of the virus between the owner and the dog is suspected [15]. Moreover, when infected, most dogs are asymptomatic, produce limited titers, and have a reduced duration of viral shedding [13], and in some cases, even after the experimental inoculation, although seroconversion was observed, no virus could be isolated [16]. Decaro et al. (2020) described a case of a dog infected with SARS-CoV-2 due to close exposure to an infected owner. Interestingly, although the dog did not show any clinical signs, the virus was identified in the first 3 days after the owner had a positive PCR test [17]. We performed SARS-CoV-2 screening 4 weeks after the first clinical signs, and both tests failed to identify the virus. The virus identification is rarely successful, and the recommended window for PCR testing is still unclear [13,15,16,17,18]. In this light, serological screening might be more suitable for dogs as long as the presence of specific antibodies can be seen for a period varying from 12 days to 5 months after the viral infection [19]. When the ELISA technique is used for diagnosis, two consecutive serum samples must be tested: an increase in the antisera’s titer over time is considered to be suggestive of confirming the diagnosis. On the other hand, the seropositivity to various antigenic sites of the SARS-CoV-2 virus can be shared by many other coronaviruses from other species [20], and caution must be taken when interpreting the presence of positive antisera without testing the neutralizing effect on SARS-CoV-2. In our case, the presence of specific anti-SARS-CoV-2 IgG, IgA, and IgM antibodies was observed twelve weeks after the first clinical signs. The presence of neutralizing antibodies was not investigated in the dog we examined, and no additional test was performed to see the evolution of antisera titer. However, the presence of IgM and the epidemiological context are highly suggestive of the presence of a new recent infection with SARS-CoV-2.

Thus, although the antigenic tests failed to identify the virus, the specific antibodies detection might be considered as indirect evidence of SARS-CoV-2 infection.

Although circulating AChR antibodies were not detected in the dog, it cannot be excluded that the SARS-CoV-2 infection may have triggered an autoimmune process and determined the occurrence of acquired MG.

Cases with seronegative titer for AChR antibodies are sporadically described in dogs (approximately 2% of dogs with generalized MG) [2]. Criteria used to diagnose MG in seronegative dogs include consistent clinical signs, consistent pharmacologic (positive edrophonium response) and electrophysiologic (decrement) findings, normalization of limb muscle weakness after anti-cholinesterase therapy, and repeated AChR antibodies (antibody titers should be repeated 1 to 2 months after a negative AChR antibody titer) [2]. In our case, all the criteria were fulfilled, with the exception of repeated titer for AChR antibodies, which was not performed due to glucocorticoid therapy, which might mask the antibodies titer. We substituted the edrophonium challenge test with the neostigmine test. In the absence of edrophonium, the clinical test with neostigmine appears to be a safe and viable alternative [21]. The clinical response of neostigmine is consistent, and with the exception of polymyositis, false positive responses were not reported [21]. In the dog we examined, the creatine kinase level was normal (69.7 UI/L; normal range 10–200 UI/L [11]), and no signs of muscle atrophy, myoglobinuric discharge, or independent muscular activity were noticed during electromyography. Consequently, a muscular pathology was excluded. Furthermore, a toxic exposure able to block the synapse was excluded by the normal level of cholinesterase (6168.5 U/L, normal range 3164–8164 U/L [2]).

The electrodiagnostic test was consistent with MG; besides the normal EMG, no changes were observed for electroneurography. The only feature was the decrement during the repetitive stimulation. The amplitude decrement during the repetitive stimulation test is a common feature in MG [2,3].

Usually, seronegative myasthenic dogs elicit the congenital form of MG. In the dog we examined, the clinical signs occurred at the age of 2 years, and the owner reported no previous clinical signs suggestive of MG. Interestingly, the clinical signs suggestive of a focal MG presentation (dysphagia, regurgitation) occurred a few days after the owner’s infection with SARS-CoV-2. In the past year, few reports described the occurrence of MG in human individuals secondary to SARS-CoV-2 [22,23,24,25] infection or secondary to the vaccine administration [26]. A handful of patients with *myasthenia gravis* showed exacerbation of their disease after acquiring COVID-19 [27]. Although the mechanism is still unknown, it is believed that the viral infection promotes CD4+ T cell-mediated B cell activation and synthesis of pathogenic high-affinity autoantibodies. The autoantibodies bind to the AChR and lead to impaired neuromuscular transmission and clinical manifestation of the disease [28]. To the best of our knowledge, this is the first report to describe a possible causality between SARS-CoV-2 infection and MG occurrence in dogs.

In the dog we examined, after the initiation of anti-cholinesterasis therapy, the weakness disappeared. The radiological aspect compatible with megaesophagus was still present, and minor cranial nerve deficits were observed (e.g., the blink reflex). Interestingly, an increase in cCRP was observed despite the normal CBC, suggesting the presence of a non-specific inflammatory response. Most of the human patients with MG who showed exacerbation of their disease after acquiring COVID-19 disease recovered with either intravenous immunoglobulins or steroids [27]. The addition of glucocorticoids normalized the cCRP values and might support an autoimmune etiology, even if the AChR titer was negative. Four mechanisms are proposed for seronegative AChR dogs: (1) antibodies are directed against non-AChR end-plate determinants; (2) antibodies are directed against the toxin binding site such that patients with antibodies to this site would seem to be seronegative; (3) antibodies are bound to the end plates without detectable circulating serum antibodies (antigen excess); and (4) antibodies are directed against antigenic determinants that may be lost during the AChR extraction procedure [2]. In addition, autoantibodies against the muscle-specific receptor tyrosine kinase, MuSK, might be involved, but testing them is limited and no established reference range is available in dogs [4].

After the MG occurrence, our dog was infected with *Babesia gibsoni*. Tick paralysis is incriminated as differential in acute flaccid tetraparesis in dogs [29,30]. However, in Europe, the tick vector has not been described; such cases are most common in the USA and Australia [30], but are sporadically reported in other geographical spaces, including in humans [31]. Some authors reported the occurrence of a tick paralysis-like disease in a few cases in Europe, but the clinical course differs and the etiological vector was never proven. Moreover, the symptomatology of tick paralysis is compatible with the generalized or fulminant form of MG, but no episodes of remission are observed, as in the dog we examined. Ticks may act as vectors for some bacterial diseases (e.g., borreliosis). Lyme disease is a tick vectorial disease that may cause neurological signs [32]. According to a study performed in a large number of dogs, the most common clinical signs in dogs with Lyme disease are lameness, neurological signs, nephropathy, lethargy, anorexia, and fever, with a history of tick infestation [33]. In the dog we examined, the tick infestation was indirectly documented by the blood smear examination. However, no other clinical signs except weakness were reported in the history, and the serum *Borrelia* IgG and IgM were negative.

Acquired MG may be associated with hypothyroidism and hypoadrenocorticism, but has also been reported as a paraneoplastic syndrome associated with thymoma, osteosarcoma, and cutaneous lymphoma [2] and trombocitopenia [34]. In the dog we examined, all the clinical and paraclinical exams performed upon presentation to our service excluded the above-mentioned potential associated conditions. In some dogs, the presence of *Babesia* may induce the occurrence of some autoimmune conditions, such as autoimmune hemolytic anemia [35]. Interestingly, evidence supports the statement that once the anti-parasitical medication is started, there is no need for immunosuppression to treat hemolytic anemia [35]. In our dog, the babesiosis was diagnosed two weeks after the occurrence of clinical signs suggestive of focal MG. We suspect that the infection with *Babesia* occurred immediately after the quarantine period, once the regular outdoor physical activity was reinitiated. After the anti-parasitical treatment was applied, no signs of hemolytic anemia were found on multiple CBC and cytological examinations of the blood smears. Despite the favorable response to anti-cholinesterasic therapy, MG is generally associated with a reserved prognosis. Aspiration pneumonia and respiratory failure remain frequent causes of death from MG in both dogs and cats [1]. Specific care is needed to prevent aspiration pneumonia, especially in patients with resistant megaesophagus (elevation of water and food bowls). Regular thoracic X-rays, CBC, and cCRP are recommended for monitoring such patients and to adapt the therapeutical approach [36,37].

## 4. Conclusions

Here, we described a dog with seronegative MG and concomitant SARS-CoV-2 infection due to close exposure to an infected owner. Our data contribute to the limited number of reports which document the viral passage from owners to pets and underline the role of serological screening for specific anti-SARS-CoV-2 antibodies as an indirect marker of infection, especially when viral detection fails in PCR tests. Although circulating AChR antibodies were not detected in the dog we examined, we cannot rule out that the virus might act as a trigger in developing autoimmune conditions such as MG through alternative mechanisms, analogously to what is described in humans. The combined pyridostigmine and prednisolone therapy offered an effective clinical response despite its limited effect on the megaesophagus.

## Figures and Tables

**Table 1:** Specific anti-SARS-CoV-2 antibodies profile 12 weeks after the debut of clinical signs.

Anti-SARS-CoV-2 Specific Antibodies	IgG (U/mL)	IgA (U/mL)	IgM (U/mL)
Anti-SARS-CoV-2—nucleocapsid (N) protein	8.521	8.490	142.050
Anti-SARS-CoV-2–spike S1 subunit receptor binding domain RBD	17.638	7.858	144.000
Anti-SARS-CoV-2—S2 unit of spike (Spike S2)	24.968	7.679	131.563
Anti-SARS-CoV-2—envelope (E) protein	19.730	6.704	132.002
Anti-ACE2—(antibodies of angiotensin—converting enzyme 2)	19.287	7.564	139.756
Anti-PL pro—(papain-like protease)	9.9710	9.538	163.163

## Data Availability

All study data are presented in the article.

## Supplementary Materials

The following supporting information can be downloaded at: https://www.mdpi.com/article/10.3390/vetsci9070318/s1, Figure S1: IgG and IgM results for Borrelia. Figure S2: AChR antibodies titer. Videos S1–S3: Clinical examination before neostigmine clinical challenge. Video S4: Clinical examination after neostigmine clinical challenge.
