# Supplementary material for: Seronegative Myasthenia Gravis with Concomitant SARS-CoV-2 Infection in a Dog
Source: Vet Sci. 2022 Jun 24;9(7):318. doi: 10.3390/vetsci9070318 (PMC9323121; doi:10.3390/vetsci9070318)
Supplement: Supplementary file 1 [file vetsci-09-00318-s001.zip › Figure S1-IgG and IgMBorrelia.pdf]

LABOKLIN

LABOR FÜR KLINISCHE DIAGNOSTIK GMBH & CO. KG

[redacted]
[redacted] Iasi
Rumänien

LABOKLIN GmbH&CoKG
Steubenstraße 4
DE-97688 Bad Kissingen
Fax-Nr.: +49 971 68546
Tel.: +49 971 72020

Patient identification:	Dog	Female	* 01-03-2020
	Cross Breed Ursa		
Owner / Animal-ID:	[redacted]		
Type of sample:	1x centrifuged sample		
Date sample was taken:	10-03-2022		

Borrelia-Antibodies (ELISA):

Borr IgG (EIA)	**1.1**	VE	< 8
Borr IgM (EIA)	**4.8**	VE	< 8

Interpretation:
IgG-and IgM values below 8 VE are considered negative, above 12 VE positive.Values in between are questionable.Sero conversion takes place approx. 30 days p.i.(first IgM, then IgG).Positive IgG results do not always correlate with acute borreliosis,titers can persist over a long period of time.
Additional tests:
Causative organism isolation (PCR) from synovia, ticks or affected tissue; Western blot assay for clarification of questionable serological results and for the differentiation of vaccination and infection.

*** END of report ***

TÄ Fr. Dr. Stieger
Abt. klin.Labordiagnostik
