# Supplementary material for: Seronegative Myasthenia Gravis with Concomitant SARS-CoV-2 Infection in a Dog
Source: Vet Sci. 2022 Jun 24;9(7):318. doi: 10.3390/vetsci9070318 (PMC9323121; doi:10.3390/vetsci9070318)
Supplement: Supplementary file 1 [file vetsci-09-00318-s001.zip › Figure S2 - AChR antibodies titer.pdf]

LABOKLIN

LABOR FÜR KLINISCHE DIAGNOSTIK GMBH & CO. KG

[illegible]
[illegible]
[illegible]
[illegible] .s1
Rumänien

LABOKLIN GmbH&CoKG
Steubenstraβe 4
DE-97688 Bad Kissingen
Fax-Nr.: +49 971 68546
Tel.: +49 971 72020

Patient identification:	Dog	Female	* 01-03-2020
	Crossbreed Ursa		
Owner / Animal-ID:	[illegible]		
Type of sample:	Serum		
Date sample was taken:	21-03-2022		

Parameter	Value	Reference value
**Acetylcholinreceptor-antibodies:**		

The test detects circulating antibodies to the Acetylcholinreceptor.
Positive results confirm the diagnosis of Myasthenia Gravis.
Therapy includes treatment with anticholinesterase and immunosuppressive agents.

Cell line TE671:	**50**	< 400
Cell homogenate:	**50**	< 300

Interpretation
Cell line TE671 < 400 and cell homogenate < 300: negative
Cell line TE671 = 400 and cell homogenate = 300: borderline value
Cell line TE671 > 600 and cell homogenate > 450: positive

*** END of report ***
